# Neuroligin 3 Regulates Dendritic Outgrowth by Modulating Akt/mTOR Signaling

**DOI:** 10.3389/fncel.2019.00518

**Published:** 2019-11-29

**Authors:** Jing Xu, Yong-lan Du, Jing-wei Xu, Xiao-ge Hu, Lin-fan Gu, Xiu-mao Li, Ping-hong Hu, Tai-lin Liao, Qiang-qiang Xia, Qi Sun, Lei Shi, Jian-hong Luo, Jun Xia, Ziyi Wang, Junyu Xu

**Affiliations:** ^1^Department of Rehabilitation of the Children’s Hospital, Zhejiang University School of Medicine, National Clinical Research Center for Child Health, Hangzhou, China; ^2^Department of Neurobiology, NHC and CAMS Key Laboratory of Medical Neurobiology, Zhejiang University School of Medicine, Hangzhou, China; ^3^Key Laboratory of Tumor Molecular Diagnosis and Individualized Medicine of Zhejiang Province, Zhejiang Provincial People’s Hospital, People’s Hospital of Hangzhou Medical College, Hangzhou, China; ^4^Zhejiang University-University of Edinburgh Institute, Jiaxing, China; ^5^Department of Orthopaedics, The Second Affiliated Hospital, School of Medicine, Zhejiang University, Hangzhou, China; ^6^JNU-HKUST Joint Laboratory for Neuroscience and Innovative Drug Research, Jinan University, Guangzhou, China; ^7^ Division of Life Science, The Hong Kong University of Science and Technology, Clear Water Bay, Hong Kong; ^8^Division of Biomedical Engineering, The Hong Kong University of Science and Technology, Clear Water Bay, Hong Kong; ^9^State Key Laboratory of Molecular Neuroscience, The Hong Kong University of Science and Technology, Clear Water Bay, Hong Kong

**Keywords:** neuroligin 3, Akt/mTOR, dendritic outgrowth, PTEN, MAGI-2

## Abstract

Neuroligins (NLs) are a group of postsynaptic cell adhesion molecules that function in synaptogenesis and synaptic transmission. Genetic defects in neuroligin 3 (NL3), a member of the NL protein family, are associated with autism. Studies in rodents have revealed that mutations of NL3 gene lead to increased growth and complexity in dendrites in the central nervous system. However, the detailed mechanism is still unclear. In our study, we found that deficiency of NL3 led to morphological changes of the pyramidal neurons in layer II/III somatosensory cortex in mice, including enlarged somata, elongated dendritic length, and increased dendritic complexity. Knockdown of NL3 in cultured rat neurons upregulated Akt/mTOR signaling, resulting in both increased protein synthesis and dendritic growth. Treating neurons with either rapamycin to inhibit the mTOR or LY294002 to inhibit the PI3K/Akt activity rescued the morphological abnormalities resulting from either NL3 knockdown or knockout (KO). In addition, we found that the hyperactivated Akt/mTOR signaling associated with NL3 defects was mediated by a reduction in phosphatase and tensin (PTEN) expression, and that MAGI-2, a scaffold protein, interacted with both NL3 and PTEN and could be a linker between NL3 and Akt/mTOR signaling pathway. In conclusion, our results suggest that NL3 regulates neuronal morphology, especially dendritic outgrowth, by modulating the PTEN/Akt/mTOR signaling pathway, probably *via* MAGI-2. Thereby, this study provides a new link between NL3 and neuronal morphology.

## Introduction

Neuroligins (NLs) are a group of postsynaptic cell adhesion molecules that play a crucial role in synaptogenesis and synaptic transmission (Scheiffele et al., 2000; Dean et al., 2003; Chih et al., 2005). Numerous studies have linked genetic deficits of NLs, especially neuroligin 3 (NL3), to autism spectrum disorders (ASDs) (Jamain et al., 2003; Pardo and Eberhart, 2007). The mice incorporated with NL3 R451C mutation, which is associated with human mental retardation and autism, exhibits impaired social interactions but enhanced spatial learning abilities (Tabuchi et al., 2007). Moreover, NL3 knockout (KO) mice or rats exhibit a series of symptoms of ASD, including increase in motor activity, a lack of social novelty preference, perseverative behaviors, and deficit of olfaction (Radyushkin et al., 2009; Hamilton et al., 2014; Rothwell et al., 2014). However, the detailed cellular signaling pathways underlying the defects caused by malfunction of NL3 are still unclear.

Early neurodevelopment including outgrowth of dendrites and axons is important to maintain the normal function of brains. Dendritic arborization defines the degree of information integration and induction of synaptic plasticity (Hausser et al., 2000). Developmental abnormalities of neuronal dendrites, either over- or under-development, were verified to contribute to multiple mental disorders, including ASD (Kwan et al., 2016; Montani et al., 2017; Dang et al., 2018). Different molecular mechanisms have been indicated to regulate the dendritic outgrowth in the past few decades, including neurotrophic factors (McAllister et al., 1995, 1997) and neurotrophic factor stimulated signaling pathways, such as the mammalian target of rapamycin (mTOR) pathway (Jaworski et al., 2005; Kumar et al., 2005; Urbanska et al., 2012) and extracellular signal-regulated kinases (ERK) pathway (Alonso, 2004; Zhang et al., 2014). Notably, substantial papers have demonstrated that imbalanced mTOR signaling pathway and protein expression would give rise to various autism-like behaviors. The *Tsc1* ± and *Tsc2* ± mice, with decreased suppression of mTOR signaling, i.e., increased mTOR signaling, present aberrant social behaviors which could be reversed by rapamycin treatment (Goorden et al., 2007; Ehninger et al., 2008; Sato et al., 2012). Mouse models with deletion of *Pten* in forebrain neurons, leading to overactive Akt/mTOR signaling, exhibit macrocephalus, seizures, and abnormal social interaction (Kwon et al., 2006). Besides, alternations of downstream components of mTOR pathway, such as KO of *4E-BP2* and overexpression of eIF4E, factors involved in protein translation, also result in social disorder and repetitive behaviors (Gkogkas et al., 2013; Santini et al., 2013). Collectively, the studies of these monogenic mutated mouse models seemed to indicate a tight connection between autism and mTOR signaling pathway, naturally raising a question that whether NL3-related autism model is also associated with this pathway. On the other hand, interestingly, abnormal dendritic growth has been recently reported in neurons with NL3 malfunction: there were a significantly greater number of dendritic branch points in pyramidal neurons of the stratum radiatum of the hippocampus of NL3 R451C knockin mice (Etherton et al., 2011). In addition, an increased axonal growth in climbing fibers of NL3 cerebellar-conditioned KO mice has been observed, which led to an invasion of synaptic terminals into the distal molecular layer and increased climbing fiber synaptic transmission (Baudouin et al., 2012). However, whether abnormal dendritic growth also occurs in NL3-deficient mice and whether NL3 is involved in the molecular pathways regulating dendritic outgrowth, such as mTOR pathway, are still unknown.

In the present study, we employed a lentivirus-based NL3 shRNA and the ASD mouse model with NL3 KO, to examine the relationship between NL3 and mTOR signaling pathway and their roles in the neuronal morphology. We show that NL3 regulates the outgrowth of neuronal dendrites by modulating Akt/mTOR signaling pathway, and the association between NL3 and Akt/mTOR signaling pathway is mediated by phosphatase and tensin (PTEN), probably *via* MAGI-2,a membrane associated guanylate kinase previously known to bind with NL1 (Hirao et al., 1998) and NL2 (Sumita et al., 2007).

## Materials and Methods

### Animals

All procedures were performed in accordance with the National Institutes of Health Guidelines for the Care and Use of Laboratory Animals and approved by the Animal Advisory Committee at Zhejiang University. NL3 KO mice were purchased from the Jackson Laboratory (008394) and housed at the Animal Facility of Zhejiang University under a 12-h light/dark cycle and had access to sufficient food and water. Embryonic day 17 (E17) mice, born by female heterozygous parent, were used for primary cortical neuron cultures after genotyping analysis. Embryonic day 18 (E18) Sprague–Dawley rats were purchased from Shanghai SLAC Laboratory Animal Co., Ltd. and used for primary hippocampal neuron cultures.

### Plasmids

Neuroligin 3 shRNA constructs were generated by inserting shRNA double-strand DNAs into the pSuper vector (a gift from Dr. Ip, Hong Kong University of Science and Technology) and then subcloned into the modified pFUGW vector for virus generation. The HIV-1 packing vector Δ8.9 and the VSVg envelope glycoprotein plasmid were gifts from Dr. C. Lois (Massachusetts Institute of Technology). The annealing primers for NL3 shRNA were the following: 5′-GATCTCC*GTAGCCTGGTCCAAATACA* TTCAAGAGA*TGTATTTGGACCAGGCTAC*TTTTTGGAAC-3′ and 5′-TCGAGTTCCAAAAA*GTAGCCTGGTCCAAATACA*TC TCTTGAA*TGTATTTGGACCAGGCTAC*GGA-3′, as used in the previous study (Xia et al., 2019). HA-tagged NL3 constructs were generated by inserting HA tag into the pRK5-NL3 plasmid. pClneoMyc rat S-SCAM alpha was a gift from Yutaka Hata and Yoshimi Takai (Addgene plasmid #40213) (Hirao et al., 1998). PDZ1/2 (amino acids 1273-2046) was cloned into pRK5-myc vector by PCR from the cDNA of a C57BL/6 mouse brain. GFP-PTEN was obtained by PCR amplification from human cDNA library and subcloning into pEGFP-C3 vector by *Sal*I and *Not*I.

### Lentivirus Generation and Infection

As previously described (Xu et al., 2016), lentiviral pFUGW constructs, the HIV-1 packing vector Δ8.9, and VSVg envelope glycoprotein plasmid were co-transfected into HEK293T cells in a 2:1.5:1 ratio by calcium phosphate DNA co-precipitation. Fresh culture medium that replaced the transfection medium at 8 h after transfection was collected at 36 h after transfection to harvest the released viral particles. Harvested medium was centrifuged at 1000 r/min at 4°C for 5 min to remove cell debris, filtered through a 0.45-μm filter, and then aliquoted and stored at −80°C. For neuron infection, the viruses were added directly into the culture media according to the titer determined for each batch of viruses.

### Cell Cultures and Transfection

Cultured hippocampal neurons were prepared from embryonic day 18 rats (Sprague–Dawley). The pregnant rats were euthanized following deeply anesthetization and the fetuses were removed from their uterus. The hippocampus were dissected out from the fetal brains and then trypsinized and triturated to single-cell suspension. Dissociated hippocampal neurons were cultured in Neurobasal Medium (Life Technologies) containing the B27 supplement (Life Technologies), 2 mM Glutamax (Life Technologies), and penicillin/streptomycin (Life Technologies). The same method was used for culturing cortical neurons from the newborn NL3 KO mice. HEK293T cells were cultured in MEM media (Life Technologies) containing 10% fetal bovine serum (Life Technologies) and penicillin/streptomycin (Life Technologies). HEK293T cells were transfected using the calcium phosphate co-precipitation method, and the medium was completely changed after 8 h.

### Antibodies and Chemical Inhibitors

The following primary antibodies were used: NL3 (self-made, generated against the amino-acids 706–825 fragment of NL3, 1:1000 for western blot, 1:100 for immunocytochemistry), MAP2 (Abcam, ab11267, 1:500 for immunocytochemistry), phospho-S6S235/236 (CST, 4858, 1:2000 for western blot), S6 (CST, 2217, 1:2000), phospho-AktT308 (CST, 2965, 1:1000 for western blot, 1:100 for immunocytochemistry), phospho-AktS473 (CST, 4060, 1:1000 for western blot, 1:100 for immunocytochemistry), Akt (CST, 9272, 1:1000), phospho-mTOR-S2448 (CST, 5536, 1:1000), mTOR (CST, 2983, 1:1000), PTEN (HuaBio, ET1606-43, 1:2000 for western blot, 1:200 for immunocytochemistry), puromycin (Millipore, MABE343, 1:10,000), β-actin (HuaBio, EM21002, 1:5000), GAPDH (Beyotime, A7016, 1:10,000), HA-tag (Cell Signaling Technology, 3724, 1:2000 for western blot, 1:500 for immunocytochemistry), and myc-tag (Sigma-Aldrich, 11667149001, 1:2000 for western blot, 1:500 for immunocytochemistry). HRP- and fluorophore-conjugated secondary antibodies were purchased from Jackson ImmunoResearch Laboratories and used 1:500 for immunocytochemistry or 1:10,000 for western blots. Rapamycin (CST, 9904) and LY294002 (CST, 9901) were prepared as 2000 × stock solutions in DMSO and used at working concentrations of 10 nM and 10 μM, respectively.

### Immunocytochemistry

Cells cultured on coverslips were fixed with 4% paraformaldehyde in phosphate-buffered saline (PBS) containing 4% sucrose for 15 min, permeabilized with 0.2% Triton X-100 for 10 min, and blocked with 10% normal donkey serum (NDS) for 1 h at room temperature. Thereafter, cells were incubated with primary antibodies in 3% NDS at 4°C overnight and fluorescent secondary antibodies at room temperature for 1 h after washing three times with PBS. Following incubation, washed coverslips were mounted using Mowiol mounting medium (w/v 24% glycerol, 9.6% Mowiol 4-88, 2.5% DABCO; v/v 36% ddH2O, 48% 0.2 M Tris pH 8.5), and imaged under an Olympus FV1000 confocal laser scanning microscope. The intensity of phospho-Akt and PTEN was measured by MetaMorph software. Neuronal morphology was analyzed by extracting and measuring MAP2-positive dendrites. We quantified soma size of the neurons by MetaMorph software. To estimate the dendritic morphology, NeuronJ, an ImageJ plugin, was used for tracing the neurites and quantifying dendritic length and arborization.

### SUnSET

Five days after infection of shNL3 lentivirus, puromycin (Sigma) at working concentration (1 μM) was added into the neuronal culture medium and incubated for 30 min. Subsequently, the treated neurons were washed twice with cold PBS and harvested for immunoblotting analysis using the 12D10 monoclonal antibody against puromycin.

### Golgi Staining

Golgi stainings were performed using an FD Rapid GolgiStain^TM^ Kit (PK401; FD Neurotechnologies, Inc.) according to the manufacturer’s instructions (Shen et al., 2016); 3-month old mice were euthanized using cervical dislocation following deeply anesthetization. Whole brains were isolated quickly from each animal, rinsed once in Milli-Q water, and sequentially immersed in impregnation solution (mixed by Solutions A and B) and Solution C. Tissues were then serially cut into sections of 120-μm thickness with a vibratome (Microm, 920120), stained with silver nitrate solution (Solutions D and E), dehydrated through descending alcohol series, and mounted with Permount lastly (Thermo Fisher Scientific). Images were acquired with a microscope (BX61; Olympus). We reconstructed the neurons by Neuronstudio and measured soma area of somatosensory cortex in the MetaMorph software by filtering puncta with area > 95 μm^2^ and length < 40 μm to define a cell body. Dendritic length and arborization were analyzed by NeuronJ and Sholl analysis, respectively, in ImageJ software.

### Co-immunoprecipitation

HEK293T cells transfected with different plasmid combinations were lyzed with 1% Triton X-100 in PBS and phosphatase inhibitor cocktail (Sigma-Aldrich) at 36 h after transfection. After centrifugation at 14,000 r/min for 25 min, the supernatant was collected and incubated with antibodies for 2 h at 4°C. Protein A beads (GE Healthcare, 17-0780-01) were then added and incubated for 2 h. The samples were eluted with 1 × SDS sample buffer after washing once with cold PBS (1% TritonX-100), twice with lysis buffer and twice again with PBS buffer, and then analyzed by SDS-PAGE and subjected to western blot analysis.

### Statistics

For each quantification, we used more than three independent experimental repeats (*n* represents experimental repeats), and 15–25 neurons were analyzed in each immunostaining experiment. The statistical analyses were conducted with IBM SPSS statistics. Band intensities of western blots were compared with one-sample *t-*test following normalized by setting each control as one. Signal intensities of immunostaining and morphological quantifications for two groups were compared with two-tailed Student’s *t*-test. Multiple pairwise comparisons were carried out by one-way ANOVA or two-way ANOVA followed by *post hoc* test. GraphPad Prism 6 was used for data display. Significance is reported as *P* < 0.05, and data were presented as mean ± standard error of the mean (SEM).

## Results

### Pyramidal Neurons of NL3 KO Mice Exhibit Morphological Changes

**FIGURE 1:**
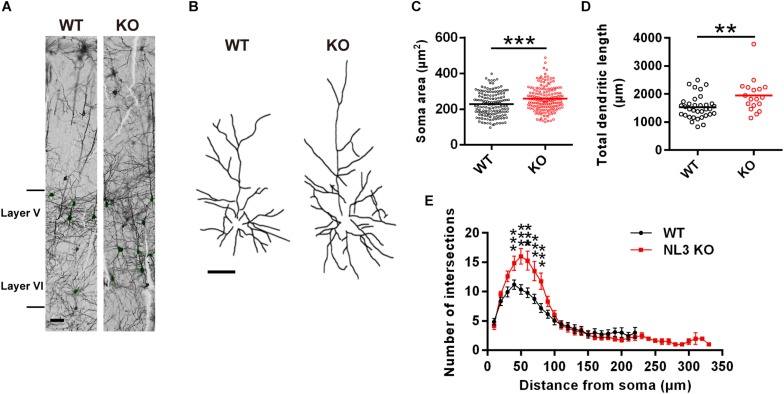
Changes in dendritic outgrowth in NL3 KO somatosensory cortex. **(A)** Golgi staining of somatosensory cortex in NL3 KO (right) and WT (left) littermates at 3 months of age. Layer V and VI were indicated. Scale bar, 50 μm. **(B)** Representative tracings of Golgi-stained pyramidal neurons in layer II/III somatosensory cortex. Left, wild type; right, NL3 KO. Scale bar, 50 μm. Quantifications of the soma area **(C)**, total dendritic length **(D)**, and sholl-analysis for dendritic arborization **(E)** of the pyramidal neurons from layer V/VI somatosensory cortex. Values represent mean ± SEM (for **C**, *n* = 147 and 170 neurons, respectively, and for **D** and **E**, *n* = 25 and 18 pyramidal neurons, respectively, from three pairs of brains; ^∗∗∗^*p* < 0.001, ^∗∗^*p* < 0.01, unpaired two-tailed Student’s *t*-test).

To examine if there were any morphological changes in neurons with NL3 deficiency *in vivo*, we conducted Golgi staining to evaluate the growth of neuronal somata and dendrites in NL3 KO mice. As a result, structural changes were observed in the somatosensory cortex from 3-month-old KO mice. The V/VI cortical layers, containing the largest percentage of pyramidal neurons in the mouse cortex, showed larger soma size compared to WT mice (Figures 1A,C). In order to reconstruct well-defined single neurons to assess the dendritic structures, we selected pyramidal neurons from layer II/III cortex instead of densely arranged neurons from layer V/VI to be outlined (Figure 1B). The length of the dendrites from KO mice, measured by Neuron J, was significantly greater than that of WT mice (Figure 1D). Sholl analysis on the tracings showed that the number of dendritic intersections was increased in the KO mice, indicating a higher dendritic complexity (Figure 1E). Taken together, the NL3 KO mice exhibited abnormal morphological changes including somatic enlargement and excessive dendritic outgrowth.

### NL3 Knockdown Activates mTOR Signaling Pathway and Affects Dendritic Outgrowth

**FIGURE 2:**
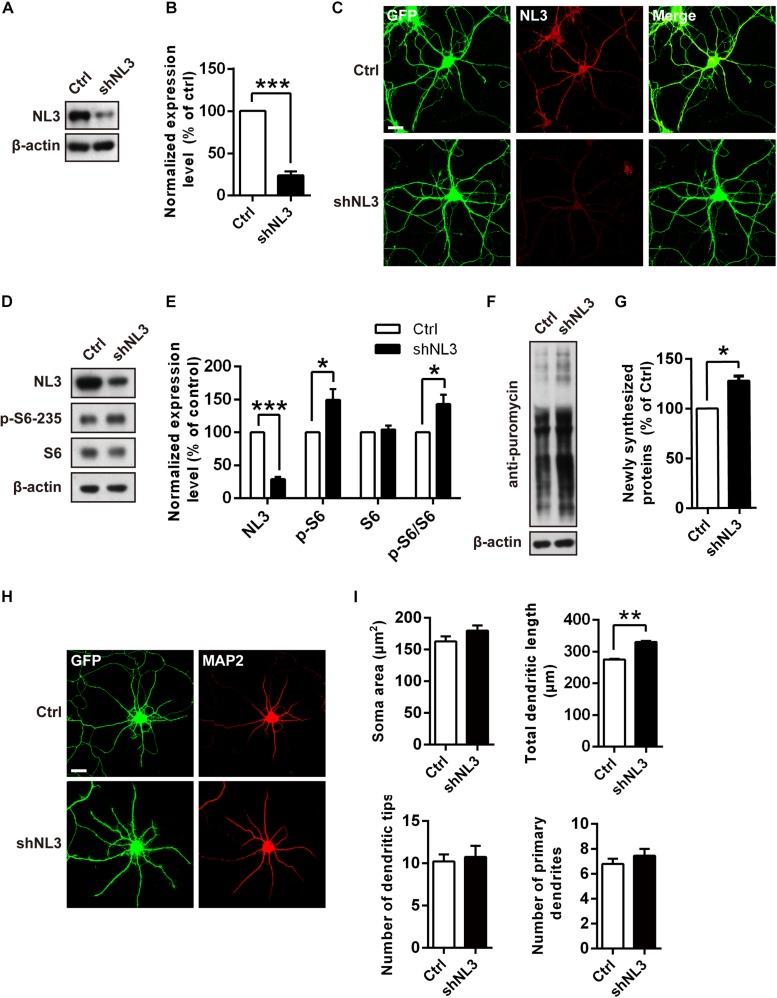
Overactivation of mTOR signaling and abnormal dendritic outgrowth in neurons with NL3 knockdown. **(A)** Western blots of cultured rat hippocampal neurons infected by shNL3-expressing lentivirus or the control lentivirus at DIV5, and harvested at DIV10. Ctrl, control; shNL3, NL3-shRNA. **(B)** Quantification of the NL3 expression as in **A**. NL3 expression of shNL3 infected neurons was reduced by 76% compared with control. Values represent mean ± SEM normalized to control (*n* = 6 independent experiments; ^∗∗∗^*p* < 0.001, one-sample *t*-test). **(C)** Immunostaining showed that the NL3 expression was enormously reduced in neurons infected by shNL3 lentivirus. Cultured rat hippocampal neurons infected with related lentivirus at DIV5 were immunostained at DIV10 using NL3-specific antibodies. Scale bar, 20 μm. **(D)** NL3 knockdown leads to hyperphosphorylation of S6. Immunoblots of cultured rat hippocampal neurons infected by shNL3-expressing lentivirus or the control lentivirus at DIV5, and harvested at DIV10. Ctrl, control; shNL3, NL3-shRNA. **(E)** Quantifications of the levels of NL3 and S6 phosphorylation as in **D**. The phosphorylated S6 protein level of the NL3 knockdown group was higher than that of the control group. Values represent mean ± SEM, normalized to control (*n* = 5 independent experiments; ^∗∗∗^*p* < 0.001, ^∗^*p* < 0.05, one-sample *t*-test). **(F)** NL3 knockdown promotes protein synthesis in neurons. Immunoblots of cultured rat hippocampal neurons infected with shNL3-expressing or control lentivirus at DIV5, incubated with puromycin at DIV10, and examined for newly synthesized proteins using anti-puromycin antibody. **(G)** Quantification of blots as in **F**. The expression level of puromycin-labeled proteins from neurons with NL3 knockdown was higher than that from control neurons. Values represent mean ± SEM, normalized to control (*n* = 3 independent experiments; ^∗^*p* < 0.05, one-sample *t*-test). **(H)** NL3 knockdown affects the outgrowth of dendrites. Photomicrographs of cultured rat hippocampal neurons at DIV6 which were infected with lentivirus co-expressing GFP and shNL3, or vector control at DIV1. GFP signal indicates successful viral infection. Green, GFP; red, MAP2. Scale bar, 20 μm. **(I)** Quantifications of soma size, total dendritic length, and numbers of dendritic terminals and primary dendrites based on MAP2 signals. The neurons in the NL3 knockdown group showed significantly longer dendrites. Values represent mean ± SEM (*n* = 3 independent experiments; ^∗∗^*p* < 0.01, paired two-tailed Student’s *t*-test).

Previous studies have suggested that dysfunction of mTOR signaling pathway, especially its hyperactivity, exists in several mouse models of ASD (Crino et al., 2006; Sharma et al., 2010; Zhou and Parada, 2012). mTOR-dependent activation of the translation machinery is essential for the upregulation of local protein synthesis in neuronal dendrites (Takei et al., 2004). Such regulation in mRNA translation was mainly conducted *via* the substrates of mTOR complex 1 (mTORC1), including both the p70 ribosomal S6 protein kinases 1 and 2 (S6K1/2) and the eukaryotic initiation factor 4E-binding proteins (4E-bps) (Burnett et al., 1998; Gingras et al., 1999; Takei et al., 2004). We wondered whether NL3 deficiency also affects the activity of mTOR signaling pathway, especially the S6K activity. To verify this hypothesis, we conducted RNAi-based downregulation of NL3 expression in cultured rat hippocampal neurons and then examined the activity of mTOR signaling pathway. Immunoblotting analysis confirmed the expression level of endogenous NL3 in shNL3-expressing viruses infected neurons had reduced to 23.8% of the control level (Figures 2A,B). Immunostaining against NL3 also showed an apparent decreased expression of endogenous NL3 in the infected neurons (Figure 2C). Thereafter, NL3 knockdown was conducted in cultured rat hippocampal neurons at days *in vitro* 5 (DIV5), and the activity of mTOR signaling pathway was determined 5 days later by analyzing the phosphorylation level of S6 ribosomal protein (Ser235/236), the catalytic substrate of S6K (Jeno et al., 1988). Results from western blot assays showed that the phosphorylation level of S6 was significantly increased in neurons with NL3 knockdown (Figures 2D,E), indicating that reduction of NL3 protein enhances the activity of mTOR signaling pathway in cultured hippocampal neurons.

Since the phosphorylation of ribosomal protein S6 regulates the translation process in protein synthesis, we next examined whether the reduction of NL3 expression also affected protein synthesis in neurons by performing the surface sensing of translation (SUnSET) assay (Schmidt et al., 2009), in which puromycin, an analog of the aminoacylated tRNA, was incorporated into the nascent polypeptide chain and detected by puromycin antibody to reflect the rate of mRNA translation. Our results showed that the puromycin level of NL3 knockdown group was 27.8% higher than that of the control group (Figures 2F,G), suggesting that downregulation of NL3 accelerated basal level of protein synthesis.

Since the disturbance of both mTOR signaling activity and novel protein synthesis occurred in NL3 knockdown neurons, we wondered whether NL3 insufficiency would also result in any morphological changes in neurons. Rat hippocampal neurons infected by knockdown-related lentivirus at DIV1 were immunostained against MAP2 at DIV6, and then examined under confocal microscope for morphological analysis (Figure 2H). A 20% of increase in total dendritic length was observed in the neurons infected by shNL3-containing lentivirus, although we detected no significant differences in soma size and dendrite numbers between NL3 knockdown and control groups (Figure 2I). Taken together, our results showed that NL3 knockdown could lead to the upregulation of mTOR signaling and increased dendritic growth in neurons.

### NL3 Deficiency-Induced Activation of mTOR Pathway Is Mediated by Increased Akt Activity

**FIGURE 3:**
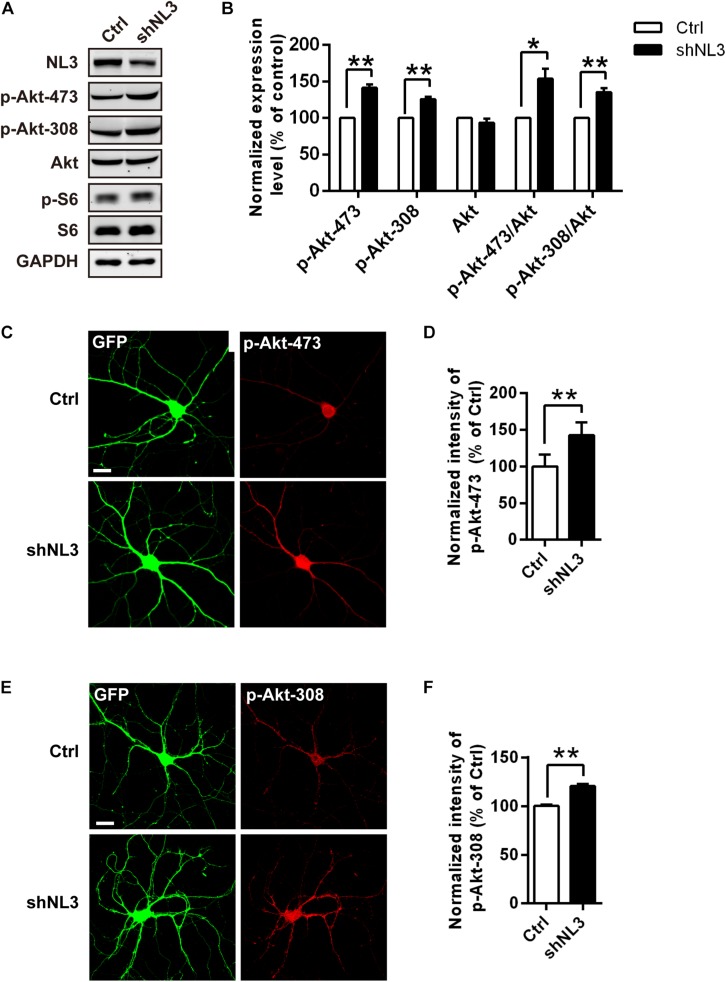
Upregulation of Akt activity in neurons with NL3 knockdown. **(A)** NL3 knockdown increased Akt phosphorylation level. Cultured rat hippocampal neurons were infected by shNL3 or control lentivirus at DIV5, and harvested for western blot analysis at DIV10. p-Akt-473, phosphorylation of Akt Ser473 residue; p-Akt-308, phosphorylation of Akt Thr308 residue. **(B)** Quantifications of the levels of NL3 and Akt Thr308/Ser473 phosphorylation as in **A**. The phosphorylation levels of both Thr308 site and Ser473 site of Akt were significantly increased in neurons with NL3 knockdown. Values represent mean ± SEM, normalized to control (*n* = 5 independent experiments; ^∗∗^*p* < 0.01, ^∗^*p* < 0.05, one-sample *t*-test). **(C,E)** NL3 knockdown increased the phosphorylation levels of both Thr308 and Ser473 sites of Akt. Cultured rat hippocampal neurons infected with related lentivirus at DIV5 were immunostained at DIV10 using phospho-specific antibodies as indicated. Scale bar, 20 μm. Quantifications of the intensities of phosphorylated Akt Ser473 **(D)** and Thr308 **(F)** as in **C** and **E**, respectively. The signal intensities of both phosphorylated Akt Ser473 **(D)** and Thr308 **(F)** were higher in the NL3 knockdown neurons than in their controls. Values represent mean ± SEM normalized to control (*n* = 3 independent experiments; ^∗∗^*p* < 0.01, paired two-tailed Student’s *t*-test).

The mTOR pathway is regulated by several upstream signaling pathways, and one of the most notable pathways is phosphoinositide-3 kinase (PI3K)/Akt. Akt activation was maximized by PI3K/PDK1-mediated phosphorylation at Thr308 site and mTOR complex 2 (mTORC2)-mediated phosphorylation at Ser473 site (Alessi et al., 1996; Sarbassov et al., 2005; Manning and Toker, 2017). We therefore examined whether Akt activity was the key factor mediating the upregulated mTOR signaling by NL3 knockdown. Phospho-specific antibodies against the Thr308 and Ser473 sites of Akt were used for both immunoblotting and immunocytochemistry assays to detect the endogenous phosphorylation level of Akt after NL3 knockdown (Figures 3A,C,E). In line with elevated p-S6 level, the phosphorylation levels of both Thr308 and Ser473 sites of Akt were significantly increased in neurons with NL3 knockdown, through either immunoblotting or immunocytochemistry assay (Figures 3B,D,F), suggesting that activation of Akt signaling is involved in NL3 knockdown-induced hyperactivated mTOR signaling.

**FIGURE 4:**
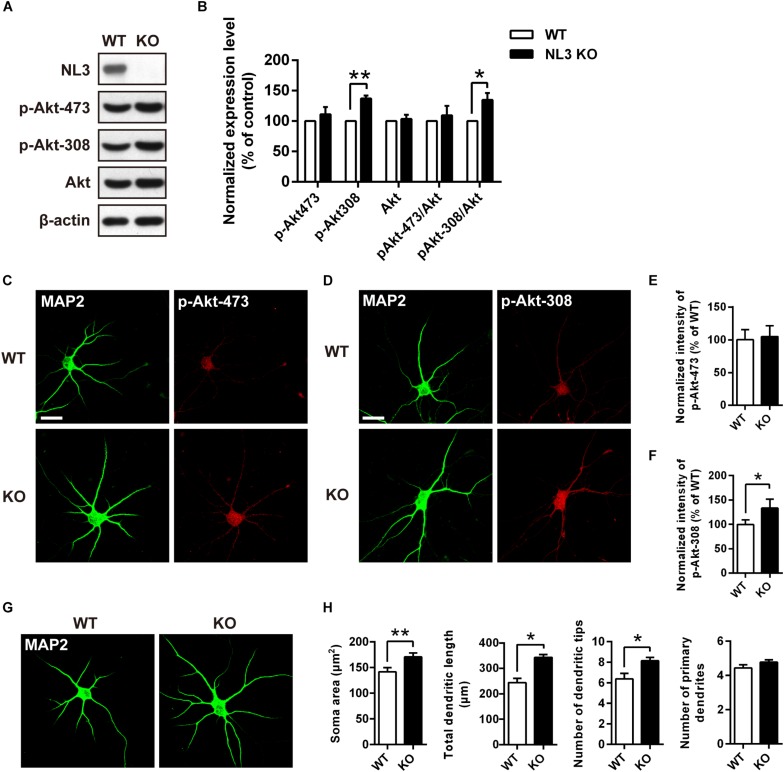
Upregulation of Akt activity and morphological changes in cultured NL3 knockout cortical neurons. **(A)** Western blot showed that Akt phosphorylation at Thr308 was increased in NL3 knockout cortical neurons. The neurons were cultured from NL3 KO newborn mice or WT littermates at P0 and harvested to be immunoblotted at DIV6. WT, wild type; KO, NL3 knockout. **(B)** Quantifications of blots as in **A**. The phosphorylation level of Akt at Thr308 sites was significantly increased in NL3 knockout neurons. Values represent mean ± SEM normalized to control (*n* = 5 independent experiments; ^∗∗^*p* < 0.01, ^∗^*p* < 0.05, one-sample *t*-test). **(C,D)** NL3 knockout cortical neurons were processed for immunocytochemistry assay at DIV6 using anti-MAP2 antibody and anti-phospho-specific antibody of Ser473 or Thr308. Scale bar, 20 μm. **(E)** Quantifications of the intensity of phosphorylated Akt at Ser473 site as in **C**. NL3 knockout neurons showed similar intensity of the phosphorylated Akt at Ser473 site with WT neurons. Values represent mean ± SEM normalized to control (*n* = 3 independent experiments; ^∗^*p* < 0.05, paired two-tailed Student’s *t*-test). **(F)** Quantifications of the intensity of phosphorylated Akt at Thr308 as in **D**. The intensity of the phosphorylated Akt at Thr308 was higher in NL3 knockout neurons than in WT neurons. Values represent mean ± SEM normalized to control (*n* = 3 independent experiments; ^∗^*p* < 0.05, paired two-tailed Student’s *t*-test). **(G)** Abnormal morphological changes in NL3 KO cortical neurons. Photomicrographs of cortical neurons cultured from NL3 knockout mice were processed for immunostaining using MAP2 antibody to label dendrites at DIV6. Scale bar, 20 μm. **(H)** Quantifications of soma size, total dendritic length, and numbers of dendritic terminals and primary dendrites. Compared with WT neurons, NL3 KO neurons showed larger soma size, longer dendritic length, and increased number of dendritic tips. Values represent mean ± SEM (*n* = 4 independent experiments; ^∗∗^*p* < 0.01, ^∗^*p* < 0.05, paired two-tailed Student’s *t*-test).

To further determine whether NL3 KO also resembles the effects of NL3 RNAi on Akt signaling and neuronal morphology, as well to exclude the off-target effect of NL3 RNAi, *in vitro* culture of cortical neurons from newborn wild-type (WT) and NL3 KO male mice was performed. Cultured neurons at DIV6 were either blotted or stained with phospho-specific antibodies of Akt (Figures 4A,C,D). Compared with the WT group, the phosphorylation level of Akt Thr308 of the NL3 KO group was significantly increased, whereas no significant change of Ser473 phosphorylation was detected either by western blot or confocal microscopy (Figures 4B,E,F). These results suggested that NL3 modulates Akt activity primarily *via* its Thr308 site.

Furthermore, we also conducted MAP2 immunostaining of cultured NL3 KO cortical neurons at DIV6 to see whether there were similar abnormalities as in NL3 knockdown neurons (Figure 4G). Statistic results showed that, compared with WT neurons, the soma size, the total dendritic length, and the number of dendritic tips of KO neurons were increased by 20, 40, and 27.6%, respectively (Figure 4H), which resembled the same morphometrical changes observed from the brain slices (Figures 1C–E). On the other hand, no significant difference in number of primary dendrites between WT and NL3 KO neurons was observed.

### Pharmacological Inhibition of Akt/mTOR Rescues Structural Abnormalities of Neurons With NL3 Deficiency

**FIGURE 5:**
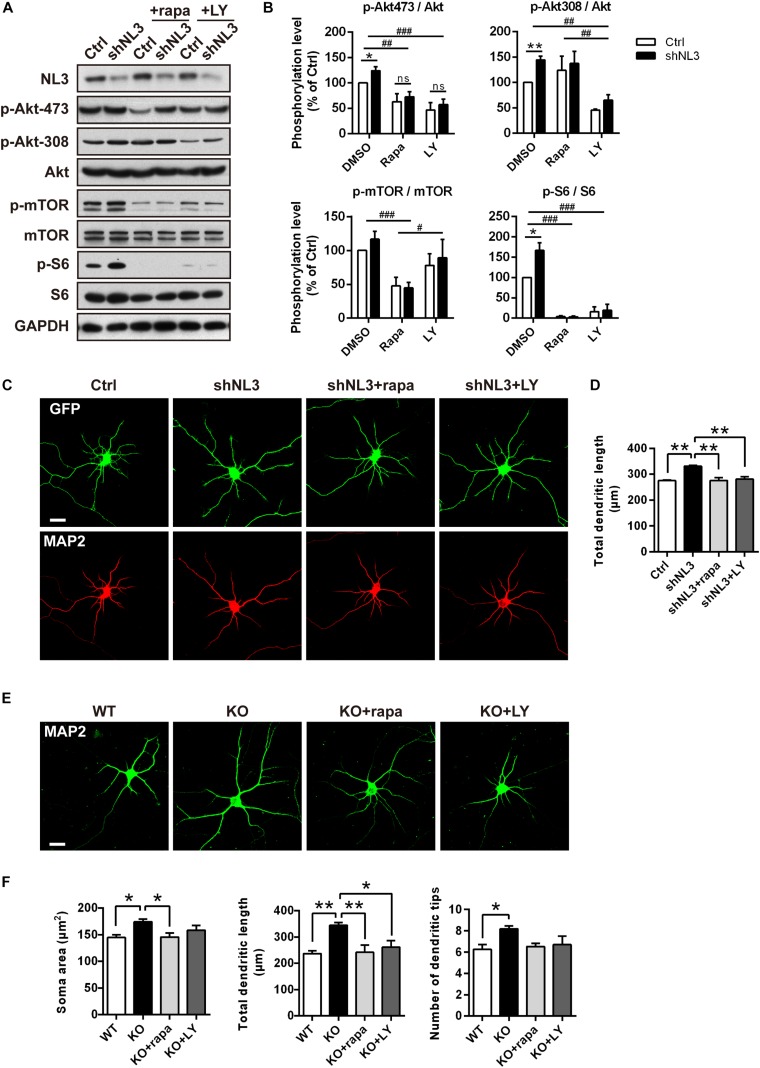
Pharmacological inhibition of mTOR signaling rescued the morphological abnormalities of neurons with NL3 deficiency. **(A)** NL3 knockdown-induced hyperactivation of Akt/mTOR signaling was inhibited by rapamycin and LY294002. Cultured rat hippocampal neurons were infected by shNL3 or control lentivirus at DIV1, incubated with DMSO, rapamycin (10 nM), or LY294002 (10 μM) for 24 h at DIV5, and then harvested and processed for western blot analysis using phospho-specific antibodies of Akt, mTOR, and S6. rapa, rapamycin; LY, LY294002. **(B)** Quantifications of the phosphorylation levels of the proteins involved in Akt/mTOR signaling pathway as in **A**. Values represent mean ± SEM, normalized to control (*n* = 5 independent experiments; ^∗∗^*p* < 0.05, ^∗^*p* < 0.05, one-sample *t*-test; ^###^*p* < 0.001,^ ##^*p* < 0.01, ^#^*p* < 0.05, two-way ANOVA with *post hoc* Bonferroni test). **(C)** Rapamycin and LY294002 rescued the neuronal morphological abnormalities induced by NL3 knockdown. Neurons with lentiviral infection at DIV1 were treated with rapamycin (10 nM), or LY294002 (10 μM) for 24 h at DIV5, and then processed for immunostaining using anti-MAP2 antibody to label dendrites. Scale bar, 20 μm. **(D)** Quantifications of the total dendritic length of the cultured neurons as in **C**. The neuronal dendrites of the NL3 knockdown group were longer than that of the control group. This increase was eliminated after either rapamycin or LY294002 treatment. Values represent mean ± SEM (*n* = 3 independent experiments; ^∗∗^*p* < 0.01, one-way ANOVA with Tukey’s *post hoc* test). **(E)** The abnormal structural changes in NL3 KO cortical neurons were reversed by rapamycin or LY294002. Cortical neurons cultured from NL3 KO mice were incubated with rapamycin (10 nM), or LY294002 (10 μM) for 24 h at DIV5, and processed for immunostaining using anti-MAP2 antibody to label dendrites. Scale bar, 20 μm. **(F)** Quantifications of the soma size, the total dendritic length, and the number of dendritic tips of the neurons as in **E**. The enlarged soma size, elongated dendritic length, and increased number of dendritic tips in NL3 KO neurons were eliminated by rapamycin treatment for NL3 knockout neurons, while LY294002 treatment rescued the enlarged soma size and elongated dendritic length in the knockout neurons. Values represent mean ± SEM (*n* = 4 independent experiments; ^∗∗^*p* < 0.01, ^∗^*p* < 0.05, one-way ANOVA with Tukey’s *post hoc* test).

Rapamycin and LY294002 are two pharmacological inhibitors of PI3K/Akt/mTOR pathway that are widely used in testing the function of this signaling pathway, which directly inhibit activities of mTORC1 (Kunz et al., 1993; Brown et al., 1994) and PI3K (Vlahos et al., 1994), respectively. To further test if downregulated Akt/mTOR signaling could effectively rescue the molecular as well as the morphometrical abnormalities in neurons caused by NL3 knockdown, rapamycin and LY294002 were added into the cultured neurons separately. Before evaluating the morphological changes of neurons, we first examined the activities of Akt/mTOR pathway after drug treatment to confirm the effectiveness of the drugs (Figure 5A). Immunoblotting analysis of cultured rat hippocampal neurons showed that the activity of Akt at the Ser473 site in the NL3 knockdown group was inhibited by both rapamycin (10 nM, 24 h) and LY294002 (10 μM, 24 h) to the same level as the control group. The decrease in phosphorylation of Akt Ser473 site, which is catalyzed by mTORC2, is consistent with the point that prolonged treatment with rapamycin inhibits mTORC2 assembly and Akt Ser473 phosphorylation (Sarbassov et al., 2006). The phosphorylation of Akt Thr308 site, meanwhile, was greatly reduced in LY294002 treated neurons due to the blocked PI3K. mTOR phosphorylation was potently inhibited by rapamycin but slightly inhibited by LY294002. Finally, the overactivation of S6, the most downstream substrate of mTOR signaling, caused by NL3 knockdown, was reversed by either rapamycin or LY294002 (Figure 5B).

We next examined the effects of rapamycin and LY294002 on the morphology of cultured neurons. Neurons infected with shNL3 at DIV1 were treated with vehicle, rapamycin, or LY294002 at DIV5 for 24 h, and then immunostained against MAP2 as the indicator of neuronal morphology (Figure 5C). Statistic results revealed that both rapamycin and LY294002 successfully eliminated the increase of total dendritic length caused by NL3 knockdown (Figure 5D).

Moreover, rapamycin and LY294002 were also added, respectively, to NL3 KO neurons at DIV5 before immunostaining (Figure 5E) to examine their effects in rescuing the morphological abnormalities after NL3 KO. Compared with WT neurons, the neurons with NL3 deletion showed larger soma size, longer dendritic length, and increased number of dendritic tips. Moreover, all these three differences were eliminated by rapamycin treatment for NL3 KO neurons, whereas only the former two were rescued by LY294002 treatment (Figure 5F). Together, these results indicate that inhibition of Akt/mTOR signaling pathway could effectively rescue the morphological abnormalities caused by NL3 deficiency.

### PTEN Mediates the Hyperactivation of Akt/mTOR Signaling Induced by NL3 Knockdown

**FIGURE 6:**
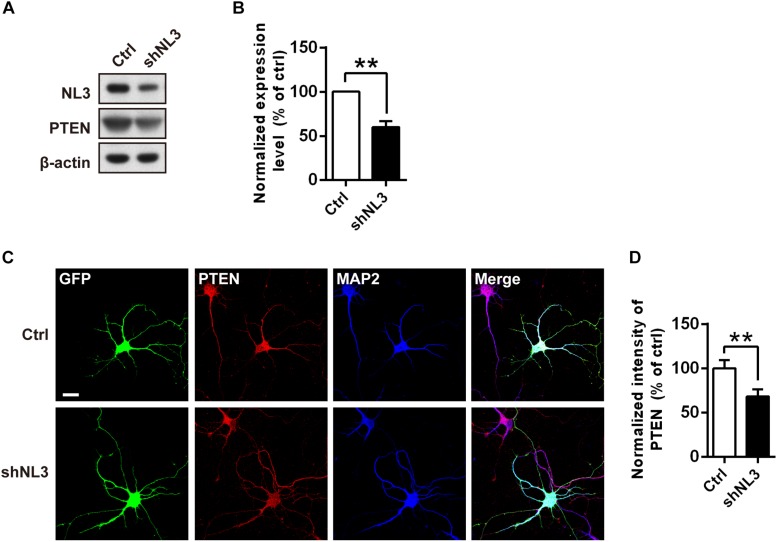
PTEN expression level is down-regulated by NL3 knockdown. **(A)** Western blot showed that NL3 knockdown reduced the protein level of PTEN. Cultured rat hippocampal neurons were infected by shNL3 or control lentivirus at DIV5, and harvested and immunoblotted at DIV10 using antibody against PTEN as indicated. **(B)** Quantification of blots as in **A**. The expression level of PTEN was significantly reduced after NL3 knockdown. Values represent mean ± SEM normalized to control (*n* = 5 independent experiments; ^∗∗^*p* < 0.01, one-sample *t*-test). **(C)** Immunostaining showed that NL3 knockdown resulted in reduced PTEN expression. Cultured rat hippocampal neurons were infected by shNL3 or control lentivirus at DIV1, and immunoreactived to PTEN (red) and to MAP2 (blue) at DIV6. Scale bar, 20 μm. **(D)** Quantification of PTEN expression level as in **C**. The PTEN expression of the NL3 knockdown group was significantly lower than control. Values represent mean ± SEM normalized to control (*n* = 3 independent experiments; ^∗∗^*p* < 0.01, paired two-tailed Student’s *t*-test).

The results above showed that the Thr308 site but not the Ser473 site of Akt exhibited a significantly increased phosphorylation level in NL3 KO neurons. It raised the possibility that the hyperactive Akt signaling was caused by PDK1 but not mTORC2. Based on these findings, we propose that NL3 may be associated with the upstream kinases which modulate the phosphorylation of the Thr308 site. As well known, PI3K phosphorylates PtdIns(4,5)P2 into PtdIns(3,4,5)P3 which prompts PDK1 to phosphorylate the Thr308 site of Akt. Conversely, the PTEN homolog inhibits the Akt activity by dephosphorylating PtdIns(3,4,5)P3 (Vander Haar et al., 2007). PTEN is found to be associated with ASD accompanying macrocephaly in both patients and murine (Butler et al., 2005; Kwon et al., 2006; Buxbaum et al., 2007; Herman et al., 2007; Orrico et al., 2009). We therefore examined the expression of PTEN in rat neurons with NL3 knockdown (Figure 6A). Western blot showed that its expression level was significantly reduced in the NL3 knockdown neurons (Figure 6B). We further examined the PTEN expression by immunocytochemical approach (Figure 6C). Result also showed a significant reduction of PTEN signal in knockdown group compared to control (Figure 6D). In order to rule out the possibility that RNAi itself had an effect on the PTEN/Akt/mTOR activity, we used a lentivirus expressing shRNA specifically targeting NL2 to test its effects on the signaling cascades by western blot (Supplementary Figure S1A). The results demonstrated that NL2 knockdown did not overactivate Akt/mTOR pathway, but also reduced the phosphorylation of Akt at Thr308 site, which indicated that the upregulation of Akt/mTOR in NL3 knockdown neurons was not induced by RNAi itself. As well, PTEN expression did not be affected by the shNL2-expressing lentivirus (Supplementary Figure S1B). These results indicate that NL3 does regulate PTEN/Akt/mTOR signaling pathway. The insufficient expression of PTEN could be a critical mediator of NL3-related hyperactivation of Akt/mTOR signaling in neurons.

### MAGI-2 Is a Potential Scaffold Protein Tethering PTEN to NL3

The decline of both NL3 and PTEN expression levels in cultured rat neurons implied a relationship between these two proteins, yet the underlying mechanism is still unknown. Notably, both NL3 and PTEN contain a C-terminal PDZ-binding motif, which raises a possibility that their interaction may be mediated by a PDZ domain or multi-PDZ domain-containing protein(s).

**FIGURE 7:**
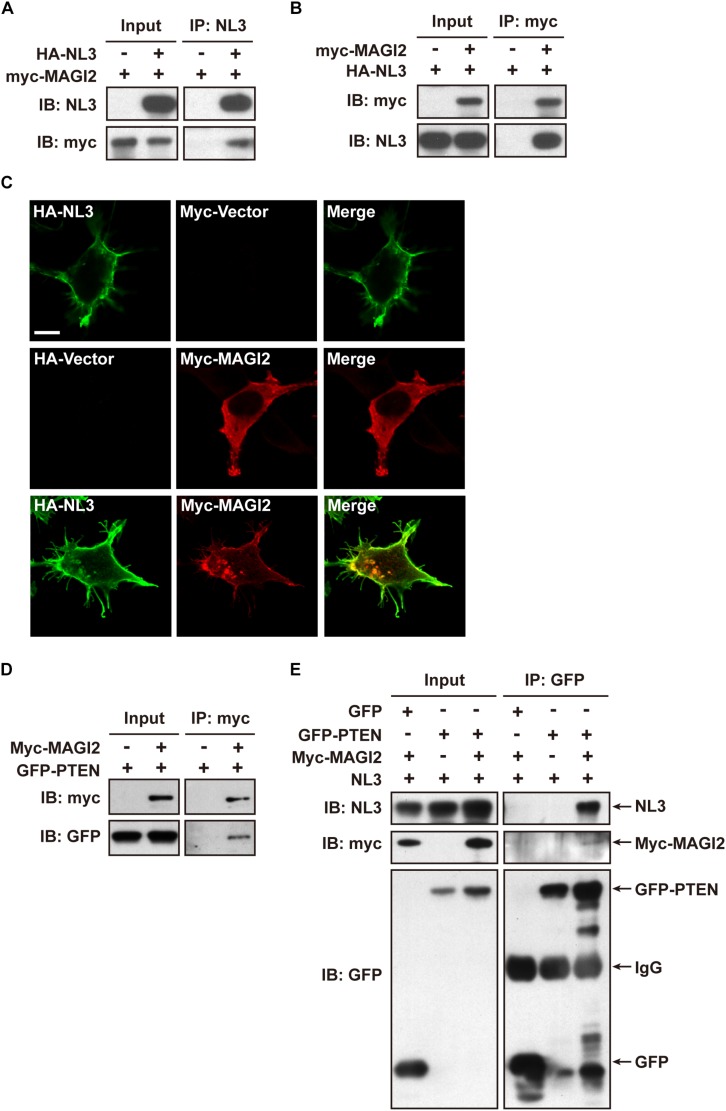
NL3 and PTEN formed a complex via MAGI-2. **(A,B)** Co-immunoprecipitation assays confirmed the interaction between NL3 and MAGI-2. HEK293T cells co-transfected with HA-NL3 and Myc-MAGI-2 plasmids or control plasmids were processed for co-IP assays using either NL3 antibody or myc antibody. Myc-MAGI-2 was pulled down together with HA-NL3 **(A)**, so was HA-NL3 by Myc-MAGI-2 **(B)**. **(C)** Myc-MAGI-2 was co-localized with HA-NL3. Photomicrographs showed that HA antibody immunostained HA-NL3 (green) is localized specifically at plasma membrane, while myc antibody immunostained myc-MAGI-2 (red) was diffusely distributed in cytoplasm in HEK293T. When myc-MAGI-2 was co-transfected with HA-NL3, it was transported to plasma membrane and co-localized with HA-NL3. Scale bar, 10 μm. **(D)** Co-immunoprecipitation assays verified the interaction between MAGI-2 and PTEN. HEK293T cells co-transfected with myc-MAGI-2 and GFP-PTEN plasmids or control plasmids were processed for co-IP assays using myc antibody. GFP-PTEN was pulled down together with myc-MAGI-2. **(E)** Co-immunoprecipitation assays in HEK293T detected that NL3 and PTEN formed a complex via MAGI-2. HEK293T cells co-transfected with NL3, GFP-PTEN, and myc-MAGI-2 or control plasmids were processed for co-IP assays using GFP antibody. NL3 and myc-MAGI-2 were pulled down together with GFP-PTEN.

Membrane associated guanylate kinase, WW, and PDZ domain containing 2 (MAGI-2, also called synaptic scaffolding molecule, S-SCAM) has been reported to interact with NL1 (Hirao et al., 1998) and NL2 (Sumita et al., 2007) *via* its PDZ1 domain at the excitatory and inhibitory synapses, respectively; the interaction between MAGI-2 and NL3 has not been verified yet. In addition, MAGI-2 is found in tumor studies to interact with PTEN *via* its PDZ2 domain and thereby stabilizes the structure of PTEN, increases its membrane trafficking, and enhances its inhibitory effect on Akt signaling and tumor pathogenesis (Wu et al., 2000; Tolkacheva et al., 2001; Nagashima et al., 2015). We hypothesized that MAGI-2 might mediate the NL3-regulated PTEN expression and membrane trafficking by simultaneously interacting with NL3 and PTEN. Hence, we first examined the interaction between NL3 and MAGI-2. Bidirectional Co-IP assays were conducted by overexpressing HA-NL3 and myc-MAGI-2 in HEK293T cells to examine the potential interaction between NL3 and MAGI-2. As expected, myc-MAGI-2 was successfully pulled down by HA-NL3, so was HA-NL3 by myc-MAGI-2 (Figures 7A,B). As well, immunostaining revealed that myc-MAGI-2 is co-localized with HA-NL3 on plasma membrane of HEK293T, whereas MAGI-2 alone was distributed diffusely in cytoplasm (Figure 7C). These results indicated that NL3 regulates the translocation of MAGI-2 to the plasma membrane through their binding. The interaction between GFP-PTEN and myc-MAGI-2, which was demonstrated in several studies (Wu et al., 2000; Jurado et al., 2010), was also confirmed through the co-IP experiment by us (Figure 7D).

We next examined whether NL3, MAGI-2, and PTEN can form a complex through the Co-IP assay. Immunoblotting result showed that, without MAGI-2 overexpression, NL3 did not show up in the immunopellet of GFP-PTEN, while in the presence of MAGI-2, NL3 could successfully co-immunoprecipitated with GFP-PTEN (Figure 7E), indicating that NL3 is able to form a complex with PTEN in presence of MAGI-2.

**FIGURE 8:**
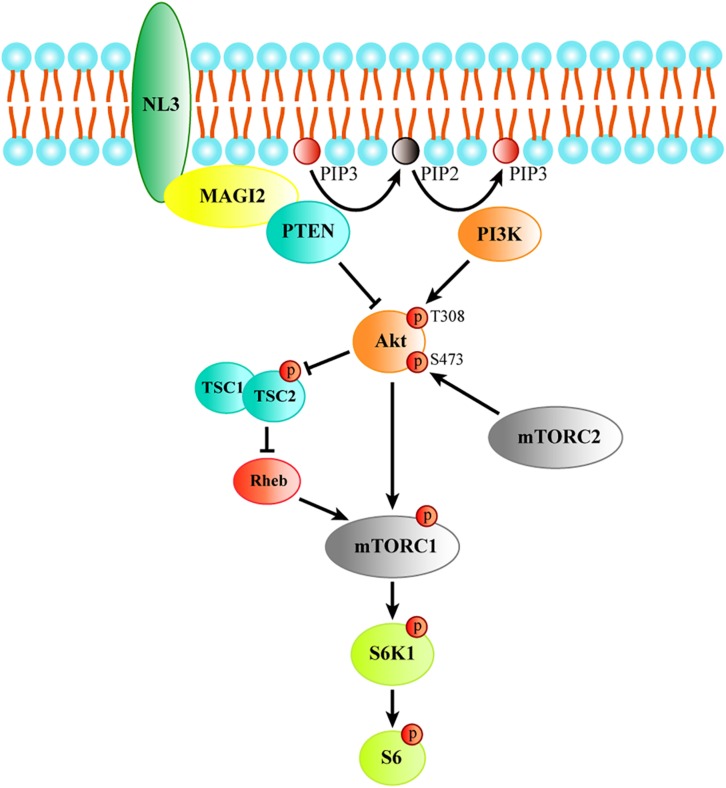
The schematic model of the process of regulating Akt/mTOR signaling by NL3. NL3 recruiting MAGI-2 to plasma membrane which could bind and stabilize PTEN to suppress the Thr308 phosphorylation of Akt catalyzed by PI3K. Akt activation is required not only the phosphorylation of Thr308 site, but also the phosphorylation of Ser473 site by mTORC2. Activated Akt is able to activate mTORC1 directly or via TSC/Rheb pathway. The activated mTORC1 phosphorylates its downstream kinase S6K1 which regulates protein synthesis by activating the substrate S6.

Together, these findings indicate that NL3 is able to recruit MAGI-2 to plasma membrane, and then regulate PTEN expression which was stabilized by binding to MAGI-2. Since PTEN works as an inhibitor of Akt/mTOR activity, we think that NL3 modulates Akt/mTOR signaling pathway through interaction with MAGI-2 and PTEN (Figure 8). Reduction or absence of NL3 expression leads to decreased trafficking of MAGI-2 to the plasma membrane, which results in unstable conformation and reduced function of PTEN. As a consequence, Akt/mTOR signaling pathway is hyperactivated in response to the reduced inhibitory effects of PTEN.

## Discussion

In summary, we have found that the defect of NL3 expression induces hyperactive Akt/mTOR signaling, which results in increased dendritic length and complexity. This aberrant outgrowth of dendrites could be reversed by both rapamycin and LY294002. Furthermore, we demonstrated that NL3 regulates Akt/mTOR signaling *via* PTEN, and MAGI-2 could function as a linker between NL3 and PTEN. Our study not only provides a detailed signaling mechanism in NL3-regulated dendritic outgrowth, but also provides a potential novel link between NL3 and the Akt/mTOR signaling pathway.

In most of our *in vitro* knockdown studies, we used dissociated hippocampal neuron culture as a neuron model because they have much uniformed cell types in culture system and therefore would be much better to elucidate the underlying mechanism of the mTOR signal transducing. However, the results we see in the KO neurons were much significant than that in the cultured knockdown hippocampal neurons as the neurons exerted greater abnormalities in soma size and dendrite extension and branching. It is probably due to the fact that KO has an immediate effect, whereas knockdown is slowly achieved (after at least 96 h). Moreover, there might be residual NL3 protein after NL3 knockdown due to the incompleteness of knockdown efficiency. In addition, the NL3-mediated regulation of neuronal morphology shows certain brain region specificities as we could observe enlarged soma and increased dendritic outgrowth in somatosensory cortex (Figure 1) but not in hippocampus (data not shown). Actually, differed function of NL3 in brain regions had been also reported in either NL3 KO or NL3 R451C knockin mice (Tabuchi et al., 2007; Etherton et al., 2011; Baudouin et al., 2012; Cao et al., 2018). Such discrepancies may due to the differed expression of NL3 or compensations of NL3 defect across brain regions.

Neuronal morphological abnormalities have been reported in several mouse models with hyperactivation of mTOR signaling pathway. For instance, mouse strains with conditional KO of PTEN showed thickened and elongated dendritic outgrowth, mossy fiber tract enlargement, and soma hypertrophy (Kwon et al., 2006). In disrupted-in-schizophrenia 1 (DISC1) KO adult mice, increase in phosphorylation level of S6 ribosomal protein, soma size, and number of primary dendrites was observed in newborn neurons (Kim et al., 2009). Here, we have demonstrated that the NL3 KO mice, as a monogenic model for ASD, had neuronal morphological abnormalities similar to these mouse models, which add to the evidence that the imbalanced PTEN/Akt/mTOR pathway leads to aberrant neuronal dendritic outgrowth in NL3-related autism model. Furthermore, in addition to the genetic defect in *Tsc1/2* and *Pten*, the lack of FMR1 expression, which leads to fragile X syndrome (FXS), an autism-associated disorder, also demonstrated hyperactive mTOR signaling both in KO mice and in FXS patients (Sharma et al., 2010; Hoeffer et al., 2012). Moreover, multiple autism-like behavioral phenotypes of *Fmr1* KO mice are able to be rescued by removal of S6K1, a downstream kinase of mTOR (Bhattacharya et al., 2012). Taken together with our findings, these studies indicated that hyperactivated mTOR signaling pathway is likely a common mechanism in different ASD models.

However, a study demonstrated that secreted NL3 promotes the growth of malignant glioma through inducing PI3K/mTOR signaling pathway (Venkatesh et al., 2015). Moreover, their recent study showed that when pediatric or adult gliomas are transplanted into NL3 KO mice, the neoplasms are unable to grow (Venkatesh et al., 2017). These studies seemed to be contrary to our findings which suggested that the reduction of NL3 expression in neurons facilitated the activation of Akt/mTOR signaling. We think that there might be three possibilities to explain this discrepancy. First, the upstream regulatory mechanism of the Akt/mTOR pathway in neurons and in glia may be different. We have observed different Akt response to NL3 deletion in neurons and glia, both of which were cultured from P0 littermate mice simultaneously (data not shown). Second, PTEN mutations that weaken its inhibitory effect on Akt/mTOR signaling are frequently found in many tumors including glioblastoma (Ohgaki et al., 2004; Parsa et al., 2007; Zheng et al., 2008; Koul, 2014), which causes more complicated modulation of Akt/mTOR pathway in glioblastoma, compared with normal neurons. Third, the function of soluble (the secreted form) NL3 could be different with its full-length protein, for example, their intra- and extra-cellular binding partners could be varied.

PTEN is a lipid phosphatase, whose substrates are mainly located on the plasma membrane. MAGI-2, a scaffold protein that anchors on the plasma membrane, in previous studies, was demonstrated to interact with the C-terminal PDZ binding motif of PTEN, and recruits PTEN to the membrane. Besides, the binding of MAGI-2 to PTEN not only stabilizes the structure of PTEN but also reduces its degradation, enhancing its inhibitory effect on Akt activity (Wu et al., 2000; Tolkacheva et al., 2001; Valiente et al., 2005). Here, we demonstrated an interaction between MAGI-2 and NL3, similar to the first report of an interaction between NL1 and MAGI-2 by Hirao et al. (1998). Our results suggested that the reduction of NL3 protein level and NL3-MAGI-2 interaction could impair the membrane trafficking and stabilization of PTEN, thus leading to the decreased amount of PTEN which hyperactivated Akt in NL3 knockdown neurons. However, the interaction between endogenous proteins under physiological conditions should be verified further, and substantial studies are required to fully elucidate this mechanism.

In clinical practice, rapamycin, also known as sirolimus, has been used as an immunosuppressive agent to prevent organ transplant rejection (Webster et al., 2006a, b). It was also used for the treatment of patients with lymphangioleiomyomatosis (LAM), a rare, cystic lung disease resulting from inappropriate activation of mTOR (McCormack et al., 2011). What is more, the application of rapamycin in the treatment for tuberous sclerosis, a multisystem genetic disease that features autistic manifestations in approximately 50% patients, has undergone phase III clinical trials (Kingswood et al., 2014; Franz et al., 2018). Our attempt of using rapamycin for treating cultured NL3 deficient neurons showed that it was well able to rescue the abnormalities of neuronal morphology. In view of the results here, we expect that rapamycin could be potentially a common targeting therapeutic agent for ASDs caused by various pathogenic genes. In addition, other proteins upstream or downstream in the mTOR signaling cascade could be potential drug targets for ASD treatment, for example, inhibition of PI3K subunit p110beta activity has been shown to improve the social activity in *Fmr1* KO mice (Gross et al., 2015), and administration of 4EGI-1, a blocker of eIF4E/eIF4G interaction, could effectively reverse the ASD-like behaviors in eIF4E transgenic mice and *4E-BP2*-KO mice (Gkogkas et al., 2013; Santini et al., 2013) and improve the context discrimination in *Fmr1* KO mice (Santini et al., 2017).

## Data Availability Statement

The datasets generated for this study are available on request to the corresponding author.

## Ethics Statement

The animal study was reviewed and approved by the Zhejiang University.

## Author Contributions

JunyuX and ZW conceived and directed the project. JingX conducted the experiments involved in this manuscript. Y-LD, J-WX, T-LL, X-GH, P-HH, and QS participated in some of the experiments. LS participated in the SUnSET assay. JingX and X-ML analyzed the data. JunX, X-ML, Q-QX, LS, J-HL, ZW, and JunyuX discussed the data. JingX, X-ML, ZW, and JunyuX wrote the manuscript.

## Conflict of Interest

The authors declare that the research was conducted in the absence of any commercial or financial relationships that could be construed as a potential conflict of interest.

## Supplementary Material

FIGURE S1 PTEN/Akt/mTOR signaling activity in neurons with NL2 knockdown. **(A)** NL2 knockdown downregulated the phosphorylation of Akt at Thr308 site, but did not affect S6 activity and PTEN expression. Cultured rat hippocampal neurons were infected by shNL2 or control lentivirus at DIV5 and harvested for western blot analysis at DIV10. **(B)** Quantifications of NL2 and PTEN expression, and Akt and S6 phosphorylation as in panel **(A)**. The phosphorylation of Akt Thr308 was significantly decreased in neurons with NL2 knockdown, but that of Akt Ser473 and S6 was not changed. The expression level of PTEN was similar with control group. Values represent mean ± SEM, normalized to control (*n* = 4 independent experiments; ^∗∗∗^*p* < 0.001, ^∗^*p* < 0.05, one-sample *t*-test).
