# Supplementary figures and images for: Neuroligin 3 Regulates Dendritic Outgrowth by Modulating Akt/mTOR Signaling
Source: Front Cell Neurosci. 2019 Nov 29;13:518. doi: 10.3389/fncel.2019.00518 (PMC6896717; doi:10.3389/fncel.2019.00518)

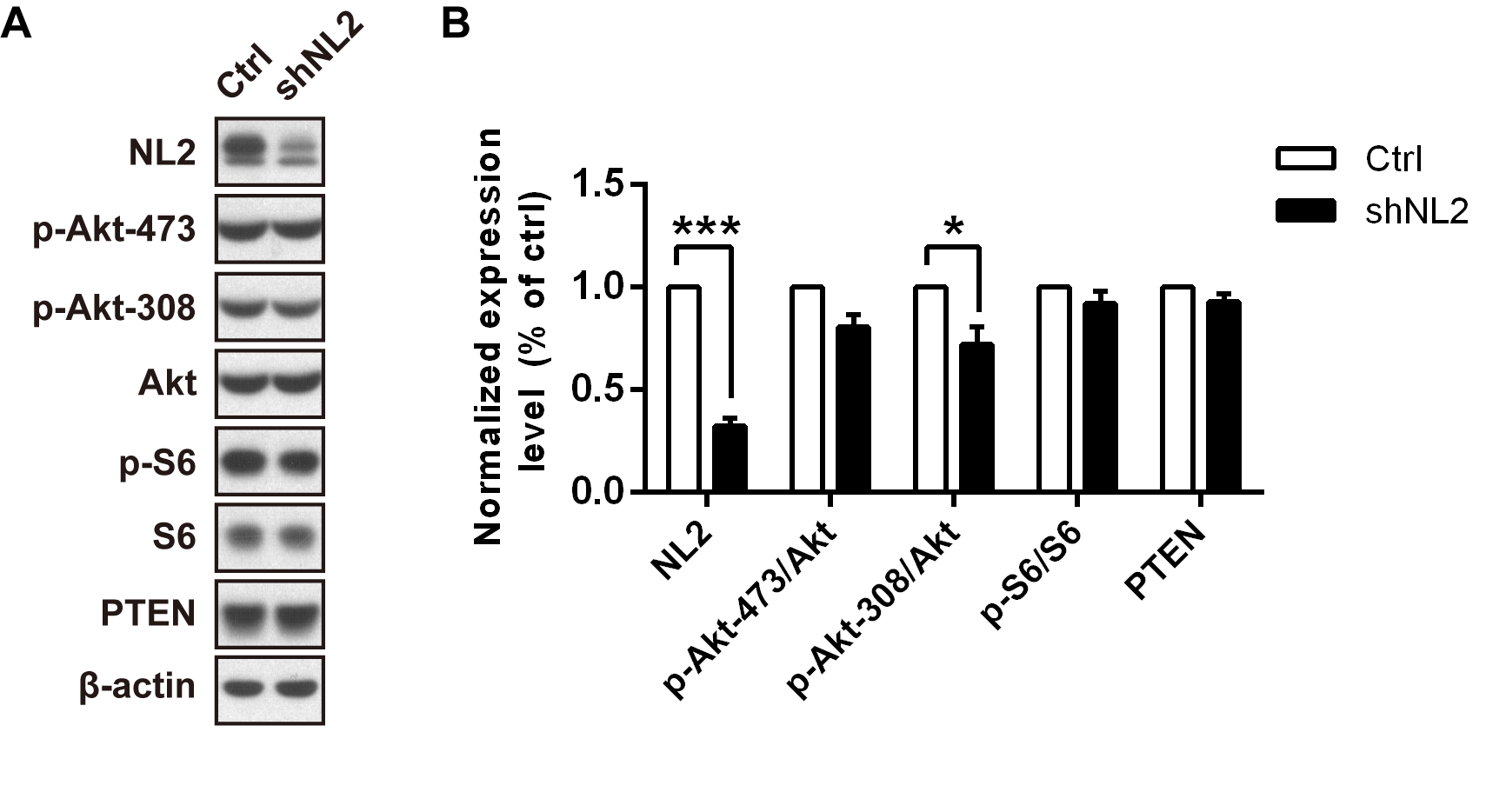

Supplement: FIGURE S1 — PTEN/Akt/mTOR signaling activity in neurons with NL2 knockdown. (A) NL2 knockdown downregulated the phosphorylation of Akt at Thr308 site, but did not affect S6 activity and PTEN expression. Cultured rat hippocampal neurons were infected by shNL2 or control lentivirus at DIV5 and harvested for western blot analysis at DIV10. (B) Quantifications of NL2 and PTEN expression, and Akt and S6 phosphorylation as in panel (A). The phosphorylation of Akt Thr308 was significantly decreased in neurons with NL2 knockdown, but that of Akt Ser473 and S6 was not changed. The expression level of PTEN was similar with control group. Values represent mean ± SEM, normalized to control (n = 4 independent experiments; ∗∗∗p < 0.001, ∗p < 0.05, one-sample t-test). [file Image_1.TIF]
